# Novel antibacterial and apatite forming restorative composite resin incorporated with hydrated calcium silicate

**DOI:** 10.1186/s40824-023-00364-z

**Published:** 2023-03-29

**Authors:** Song-Yi Yang, A Ruem Han, Ji-Won Choi, Kwang-Mahn Kim, Jae-Sung Kwon

**Affiliations:** 1 Department of Dental Hygiene, Konyang University, Daejeon, Republic of Korea; 2 Department and Research Institute of Dental Biomaterials and Bioengineering, BK21 FOUR Project, Yonsei University College of Dentistry, Seoul, Republic of Korea; 3 Department and Research Institute of Dental Biomaterials and Bioengineering, Yonsei University College of Dentistry, 50-1 Yonsei-ro, Seodaemun-gu, Seoul, 03722 Republic of Korea

**Keywords:** Restorative composite resin, White Portland cement, Hydrated calcium silicate, Antibacterial, Apatite formation

## Abstract

**Background:**

White Portland cement is a calcium silicate material. It exhibits antibacterial properties and is biocompatible. In addition, calcium silicate-based materials are known to release calcium ions and form apatite. The purpose of this study was to develop a novel bioactive restorative resin composite with antibacterial and apatite forming properties to prevent tooth caries at the interface of teeth and restorative materials, by incorporation of hydrated calcium silicate (hCS) derived from white Portland cement.

**Methods:**

To prepare the experimental composite resins, a 30 wt% light-curable resin matrix and 70 wt% filler, which was mixed with hCS and silanized glass powder were prepared in following concentrations: 0, 17.5, 35.0, and 52.5 wt% hCS filler. The depth of cure, flexural strength, water sorption, solubility, and antibacterial effect were tested. After immersion in artificial saliva solution for 15, 30, 60, and 90 days, ion concentration by ICP-MS and apatite formation using SEM-EDS, Raman spectroscopy and XRD from experimental specimens were analyzed.

**Results:**

All experimental groups showed clinically acceptable depths of cure and flexural strength for the use as the restorative composite resin. Water sorption, solubility, released Ca and Si ions increased with the addition of hCS to the experimental composite resin. Experimental groups containing hCS showed greater antibacterial effects compared with the 0 wt% hCS filler group (p < 0.05). The 52.5 wt% hCS filler group produced precipitates mainly composed of Ca and P detected as hydroxyapatite after immersion in artificial saliva solution for 30, 60, and 90 days.

**Conclusions:**

This results show that composite resins containing hCS filler is effective in antibacterial effects. hCS has also apatite formation ability for reducing gap size of microleakage by accumulating hydroxyapatite precipitates at the restoration-tooth interface. Therefore, novel composite resin containing hCS is promising bioactive resin because of its clinically acceptable physiochemical properties, antibacterial properties, and self-sealing potential for prevention of microleakage for longer usage of restorations.

**Supplementary Information:**

The online version contains supplementary material available at 10.1186/s40824-023-00364-z.

## Background

As demand for esthetic dental restorations is increasing, dental composite resins are frequently used because of their great handling and physiochemical properties and availability in various colors compared with other restoration materials. However, despite its excellent properties, poor marginal adaption, polymerization shrinkage of restorative composite resin, and marginal gaps at the interface of the restorative material and cavity walls may result in bacterial invasion, postoperative sensitivity, and subsequent inflammatory changes in the pulp [1]. This space is a niche for accumulation of biofilm and invasion by cariogenic bacteria [2]. Therefore, microleakage is a primary cause of marginal or secondary caries, which is the major cause of restoration failure [3, 4].

Secondary caries can be considered primary lesions around the restorations and also known as marginal caries. The main location of the biofilm stagnation area is cervical margin of the restorations that secondary caries can be appeared as superficial or wall lesions adjacent to restorations. To overcome this shortcoming, most of the clinical studies suggested restorations with microleakage to be removed for re-restoration rather than sealing the marginal gap.

White Portland cement is a calcium silicate-based material, which is composed of tricalcium silicate and dicalcium silicate. It exhibits antibacterial properties and is biocompatible [5]. In addition, calcium silicate-based cements are known to release calcium ions and form apatite in physiological-like phosphate solutions, such as Dulbecco’s phosphate-buffered saline (DPBS), simulated body fluid (SBF), and Hank’s balanced salt solution (HBSS) [6, 7]. In addition, apatite-like crystals at the interface of dentin and mineral trioxide aggregate (MTA) have exhibited a sealing ability by mechanical and chemical bonding [8]. In general, calcium silicate-based materials, especially hydrated calcium silicate (hCS) derived from white Portland cement, have been focused on root canal treatment such as root-end filling or apexification [9]. Especially, previous studies showed that resin composites with hCS have higher bioactivity compared to un-hydrated calcium silicate in the cariogenic environment by releasing more caries inhibit ions at the initial time.

According to Cheung, clinical techniques or dental materials have been studied to reduce the microleakage of restorations, and to improve the marginal adaptation [10]. In addition, the development of a dental composite resin with no polymerization shrinkage or slight curing expansion appears to be ideal methods for the problems arising from a restorative material which shrinks on polymerization. Alternatively, the application of a biocompatible calcium phosphate compounds produced from calcium silicate-based materials may be considered to reduce the gap size or seal the microleakage between the tooth and restoration.

To date, there has been no studies of the secondary caries prevention effects of restorative composite resin with hCS for antibacterial and self-sealing potential. Thus, study on restorative composite resin that can reduce the marginal gap by itself is needed. Accordingly, the purpose of study was to examine the antibacterial and apatite formation efficacy of composite resins containing hCS filler. This was investigated by incorporating diverse hCS contents into the light-curable resin matrix to develop a caries-preventive restorative material and analyzing its mechanical, physical, and antibacterial properties, ion release, and apatite-formation ability. The null hypothesis was that the mechanical and physical properties, antibacterial effect, and apatite-forming ability of the restorative composite resin containing hCS would not be significantly different from those of composite resin without the hCS filler.

## Methods

### Experimental groups

A light-curable resin matrix was made with 1:1 ratio of bisphenol A glycerolate dimethacrylate (Sigma-Aldrich, MI, USA) and triethylene glycol dimethacrylate (Sigma-Aldrich, MI, USA), and 0.3 wt% of camphorquinone (Sigma-Aldrich, MI, USA) by mixing with ultra-sonication for 20 min. To this mixture, 0.6 wt% of 2-(dimethylamino) ethyl methacrylate (Sigma-Aldrich, MI, USA) was added and stirred for 1 day without light disturbance. For the conventional glass filler, silane-treated filler powders (NanoFine® NF180, Schott, Landshut, Germany) composed of SiO_2_, BaO, B_2_O_3_, and Al_2_O_3_ was chosen. To prepare the hCS powder, a protocol from a previous study was used [11]. To mix the resin matrix and two types of fillers, hCS and silanized glass powder were added to the light-curable resin matrix and blended with a speed mixer (DAC 150.1 FVZ Speed mixer, Hauschild, Germany) at 3500 rpm for 5 min in a dark environment. Experimental groups were prepared using above method (Table 1), and Filtek Z250 (3 M ESPE, St. Paul, MN, USA) was used as a commercial control (CC).

**Table 1:** Composition of the experimental groups (Wt.%)

Group	0 wt% hCS	17.5 wt% hCS	35.0 wt% hCS	52.5 wt% hCS
Resin matrix	30.0	30.0	30.0	30.0
Hydrated calcium silicate	0.0	17.5	35.0	52.5
Silane-treated filler powders	70.0	52.5	35.0	17.5

### Depth of cure

According to ISO 4049 (2019), a metallic mould 4 mm in dimeter and 6 mm in height was positioned on a microscopic slide glass that was covered with a polyester film. The mould was slightly overfilled with the experimental material, without air bubbles, and then placed with a polyester film. On the top of the mould, a slide glass was placed and pressed to displace the excess material, and removed from the top of the mould. Then, the top surface was cured using a light-emitting diode curing unit (Elipar S10, 3 M ESPE Co., Seefeld, Germany) for 20 s. Cured experimental material was separated from the mould immediately after irradiation, and the uncured materials were removed using a spatula. The height of the cured materials was examined at four random areas with a micrometer (Mitutoyo, Tokyo, Japan) and the value was divided by two.

### Flexural strength

The three-point flexural strength test was conducted according to ISO 4049 (2019). A stainless steel mould with dimensions of 25 mm × 2 mm × 2 mm was placed on a slide glass covered with polyester film, and the experimental materials were dispensed in it. Another slide glass covered with polyester film was positioned on the top of the mould, and the mould was clamped and pressed to remove excess material. The experimental resin was immediately irradiated using a light-emitting diode curing unit for 30 s with overlapping areas. The ten polymerized bar-shaped specimens from each experimental group were positioned in distilled water at 37 ± 1 °C for 1 d before performing the three-point flexural strength test. The flexural strength value was obtained using a universal testing machine (Instron 5942, Instron, Norwood, MA, USA) with a crosshead speed of 1.0 mm/min until the specimens fractured. The maximum load (*F*) was observed, and the three-point flexural strength (σ) was obtained in megapascals using the formula: σ = 3*Fl*/2*bh*^2^, where *F* is the maximum load in Newtons, *l* is the 20 mm support distance, *b* is the specimen width, and *h* is the height of specimen.

### Water sorption and water solubility

Based on ISO 4049 (2019), six polymerized disk-shaped specimens from each group with a 15.0 ± 0.1 mm diameter and 1.0 ± 0.1 mm height were prepared. The diameter and height of specimens were measured to get volume (*V*), and the constant mass (m_1_) was obtained to an accuracy of 0.1 mg using a digital balance (XS105, Mettler Toledo AG, Greifensee, Switzerland). Then, the specimens were individually stored in 10 mL of distilled water at 37 ± 1 °C. After 7 d, the disk specimen was taken from distilled water, and the surface water was blotted away, then weighed (m_2_) 60 s after removal from the water. The specimen was then moved to a desiccator and weighed daily until a constant mass (m_3_) was observed. The water sorption (W_sp_, µg/mm^3^) and water solubility (W_sl_, µg/mm^3^) values were obtained using the formulas: W_sp_ = (m_2_-m_3_)/*V* and W_sl_ = (m_1_-m_3_)/*V*.

### Ion release

Eighteen polymerized specimens (25 mm × 2 mm × 2 mm) from each experimental group were prepared. To extract the ions from the specimen, a lactic acid solution was used after adjusting the pH to 4.0 by adding DL-Lactic acid (Sigma-Aldrich, Steinheim, Germany) to distilled water. Three specimens were then stored in a DL-Lactic acid solution at a ratio of 0.14 cm^3^/1 mL [12] for 1 h, 24 h, 7 d, 14 d, 30 d, 60 d, and 90 d, and the solution was exchanged at each point. After removing the specimens from the lactic acid solution at each time point, the storage solution was collected to conduct inductively coupled plasma-mass spectrometry (ICP-MS, Agilent 7900, Stockport, UK) followed by preconditioning with hydrofluoric acid to detect the calcium and silicon ions released from the specimens.

### Antibacterial **effect**

#### Strain cultivation and bacterial suspension preparation

*Streptococcus mutans* (*S. mutans*, KCTC No. 5365, KCTC, Jeollabuk-do, Korea) was incubated in the brain heart infusion broth (BHI, Difco, Sparks, MD, USA) at 37 ± 1 °C for 2 days. Using an ELISA reader (Epoch, BioTek, Winooski, VT, USA), the *S. mutans* suspension was adjusted with culture media to establish an optical density value within the range of 0.4–0.6 at 600 nm.

### Bacterial attachment to the specimen

Eleven polymerized disk-shaped specimens (10.0 ± 0.1 mm diameter and 1.0 ± 0.1 mm height) from each experimental group were prepared. The sterilized disk specimen was positioned on a 24-well plate (SPL Life Science, Gyeonggi-do, Korea), and 100 µL of the *S. mutans* suspension was dropped onto the specimen surface. The plates containing specimens were then stored at 37 ± 1 °C. After 1 day, to remove loosely attached bacteria, the specimen was gently washed with distilled water for 10 s. The disk specimen exposed to *S. mutans* was then positioned in the new 24-well cell culture plate facing up.

### Evaluation of bacteria attachment on the specimen

To visualize the attached *S. mutans* on the experimental resin surface after performing the process described in sub-section “Bacterial attachment to the specimen”, the specimen was fixed with Karnovsky’s fixative solution composed of 2% paraformaldehyde, 2% glutaraldehyde, and 0.1 M phosphate buffer solution (PBS) for 24 h. Specimen was then rinsed with 0.1 M PBS for 30 min and fixed with osmium tetroxide for 2 h. After then, the specimen was dehydrated by serial dilution using ethanol (50–100%) and dried for 2 h with a critical point dryer (Leica EM CPD300, Leica, Wien, Austria). Specimen was then coated with platinum using an ion sputter (Leica EM ACE600, Leica, Wien, Austria) and analyzed with a field emission scanning electron microscope (FE-SEM, Merlin, Carl Zeiss, Oberkochen, Germany) at an accelerating voltage of 15.0 kV.

### Bacteria viability by live/dead staining

After performing the process described in sub-section “Bacterial attachment to the specimen”, three specimens from each group were stained using a live/dead bacterial viability kit (Molecular Probes, Eugene, OR, USA). Equal volumes of propidium iodide and Syto 9 dye, which stain live and dead bacteria, respectively, were thoroughly mixed. Then, 1 mL of PBS was added to 3 µL of the propidium iodide and Syto 9 dye mixture in which each specimen attached with *S. mutans* was immersed for 20 min at 37 ± 1 °C in the dark environment. The stained *S. mutans* on the experimental resin surface was observed using a confocal laser microscope (LSM700, Carl Zeiss, Thornwood, NY, USA). Live bacteria appear green, whereas dead bacteria appear red.

### Antibacterial **activity by colony forming units (CFUs)**

After performing the process described in sub-section “Bacterial attachment to the specimen”, six specimens of each group was respectively placed in 1000 µL of culture media and sonicated for 10 min. After removing disk specimens, obtained bacterial suspension was then diluted, spread onto the BHI agar plates, and stored at 37 ± 1 °C for 2 days before CFUs counting. The relative survival rate was calculated with CFUs using the equation: relative survival rate of *S. mutans* (%) = (1 - (CFUs of remaining bacteria on the specimen / CFUs of bacterial suspension applied on the specimen)) × 100.

### Apatite **formation**

#### Preparation of the samples

Twenty-four polymerized disk-shaped specimens (10.0 ± 0.1 mm diameter and 1.0 ± 0.1 mm height) from each experimental group were prepared. Artificial saliva was made by adding 0.4411 g CaCl_2_·2H_2_O (Sigma-Aldrich, Steinheim, Germany) and 0.245 g of KH_2_PO_4_ (Sigma-Aldrich, Steinheim, Germany) in 800 mL of distilled water. To this mixture, the pH was adjusted to 7.0 by dropping 0.5 M KOH (Duksan Reagents, Ansan, Korea) [13]. Each disk-shaped specimen was immersed in 10 mL of artificial saliva solution and kept at 37 ± 1℃ for 15 d, 30 d, 60 d, and 90 d. The artificial saliva was refreshed every 15 d.

### Morphological and chemical analysis

Two disk-shaped specimens were randomly selected from the experimental groups at each time period, cross-sectioned, and polished using a polishing machine (Buehler, Lake Bluff, IL, USA) with a 1200-grit SiC abrasive paper (Deerfos, Incheon, Korea). The morphology and chemical components of the top surface and the cross section were analyzed using scanning electron microscopy-energy-dispersive X-ray spectrometry (SEM-EDS, Merlin, Carl Zeiss, Oberkochen, Germany) at a magnification of 500× under an acceleration voltage of 15.0 kV.

### Raman spectroscopy analysis

Two disk-shaped specimens were randomly selected from the experimental groups at each time period. The precipitates formed on the specimens were carefully harvested using a plastic spatula. The collected precipitates were positioned on a slide glass, and the Raman spectra were observed with a Raman spectrometer (LabRam Aramis, Horriba Jobin Yvon, France) under 50×, opening of 50 μm, laser wavelength of 532 nm, and Raman shift from 200 cm^− 1^ to 1400 cm^− 1^ [13, 14].

### XRD analysis

Two disk-shaped specimens were randomly selected from the experimental groups at each time period. The precipitates formed on the specimen surface were obtained as previously described in sub-section “Raman spectroscopy measurement”. The peaks in the diffraction spectrum of the precipitates were observed by X-ray diffraction (XRD; Ultima IV, Rigaku, Japan) using Cu Kα radiation in the 2 theta range from 20 to 60°, with a step size of 0.02.

### Cytotoxicity analysis

#### Cell culture preparation

L-929 cells, a mouse fibroblast cell line, were cultured in 1X MEM (Welgene, Gyeongsangbuk-do, Korea) cell culture medium supplemented with 1% antibiotic-antimycotic (Welgene, Gyeongsangbuk-do, Korea) and 10% fetal bovine serum (Gibco, Grand Island, NY, USA). The cells were cultured in a humidified incubator at 37 ℃ with 5% CO_2_. The adherent cells were separated with 0.05% trypsin/EDTA (Gibco, Grand Island, NY, USA) and centrifuged for 3 min followed by resuspension in cell culture medium.

### Preparation of the samples

Polymerized disk-shaped specimens (10.0 ± 0.1 mm diameter and 1.0 ± 0.1 mm height) from each experimental group were prepared. The sterilized disk specimens were extracted in a cell culture medium, 1X MEM containing 10% fetal bovine serum, with an extraction ratio of 1.25 cm^2^/mL for 24 h at 37 ℃. The eluted 1X MEM containing 10% fetal bovine serum for 24 h was used as a blank group and high density polyethylene film (Lot No.: C-212, Hatano Research Institute, Japan) was used as a negative group (NC). The L-929 cells were seeded into 96-well plates (SPL Life Science, Gyeonggi-do, Korea) and cultured in an incubator for 24 h at 37 ℃ with 5% CO_2_. Then, the cells exposed to extracts solution were cultured in a humidified incubator at 37 ℃ with 5% CO_2_. After 24 h, the extracts solution was removed and washed with PBS (Gibco, Grand Island, NY, USA).

### Methylthiazol tetrazolium (MTT) assay

To evaluate the cytotoxicity of the experimental specimen, an MTT assay was performed according to ISO 10993-5:2009 (Biological evaluation of medical devices — Part 5: Tests for in vitro cytotoxicity) and ISO 10993-12:2021 (Biological evaluation of medical devices — Part 12: Sample preparation and reference materials). At a concentration of 1 mg/mL, the thiazoly blue tetrazolium bromide (Sigma-Aldrich, St. Louis, MO, USA) was dissolved in 1X MEM without phenol red medium (Welgene, Gyeongsangbuk-do, Korea). It was then filtered using a 2 μm syringe filter (ADVANTEC, Tokyo, Japan). The 50 µL of prepared MTT solution was added to each well, and the plates were cultured in an incubator at 37 ℃ with 5% CO_2_. The MTT solution was removed after 2 h of incubation and 100 µL of isopropanol was added to each well. Then the absorbance was measured with a microplate spectrophotometer (Epoch, BioTek, Winooski, VT, USA) at 570 nm. The optical density (OD) was recorded and calculated following formulas to obtain the cell viability. Cell viability (%) = (OD570 of experimental group / OD570 of blank group) × 100.

### Statistical analysis

Acquired data on the depth of cure, three-point flexural strength, water sorption, solubility, relative survival rate of *S. mutans*, cell viability at the different hCS contents were analyzed with a one-way ANOVA test (SPSS 25, IBM Co., Armonk, NY, USA) followed by a Tukey’s post hoc test (p = 0.05).

## Results

### Depth of cure

**Fig. 1:**
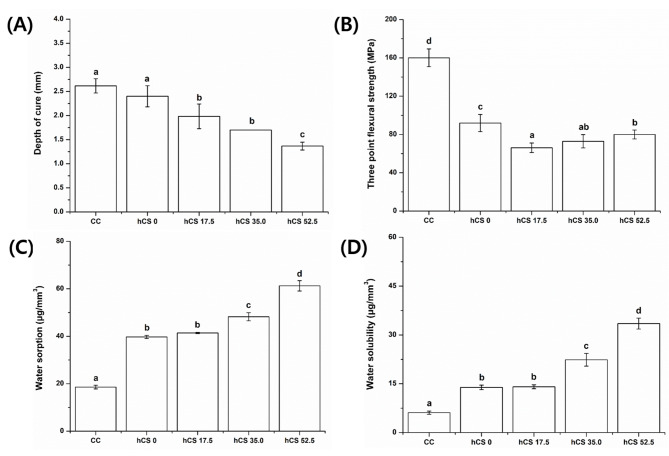
The depth of cure (A), three-point flexural strength (B), water sorption (C), and water solubility (D) of experimental groups. Each value represents the mean of the results, and the error bars represents the standard deviation of the mean. The same lower case letters indicate no significant differences among the groups (*p* > 0.05), while the different lowercase letters indicate significant differences between the experimental groups (*p* < 0.05)

The depth of cure was calculated from polymerized length of six specimens (Fig. 1A). The depth of cure in group CC was 2.62 ± 0.15 mm, the highest value among the experimental groups, and showed no significant difference from 0 wt% hCS (p > 0.05). The results revealed decreasing depth of cure as more hCS was added (p < 0.05), and there was no difference between the 17.5 wt% hCS and 35.0 wt% hCS groups (p > 0.05). The depth of cure in groups 0 wt% hCS, 17.5 wt% hCS, 35.0 wt% hCS, and 52.5 wt% hCS was 2.40 ± 0.22, 1.98 ± 0.26, 1.70 ± 0.0, and 1.37 ± 0.08 mm, respectively.

### Three-point flexural strength

Figure 1B illustrates the three-point flexural strength of the experimental groups in megapascals. The highest flexural strength was observed in group CC (160.04 ± 9.27 MPa). The flexural strength in group 0 wt% hCS was 91.92 ± 8.97 MPa, and the strength increased as more hCS was added to the resin filler. There were no significant differences between groups 17.5 wt% hCS and 35.0 wt% hCS and also between groups 35.0 wt% hCS and 52.5 wt% hCS (p > 0.05). The flexural strength of groups 17.5 wt% hCS, 35.0 wt% hCS, and 52.5 wt% hCS were 66.07 ± 4.92, 72.85 ± 6.97, and 79.99 ± 4.67 MPa, respectively.

### Water sorption and water solubility

Both water sorption and water solubility results demonstrated a similar tendency (Fig. 1C and D): the CC group exhibited minimum values, and the values increased with the addition of hCS. The values of water sorption in groups CC, 0 wt% hCS, 17.5 wt% hCS, 35.0 wt% hCS, and 52.5 wt% hCS were 18.58 ± 0.78, 39.73 ± 0.67, 41.39 ± 0.24, 48.22 ± 1.71, and 61.24 ± 2.18 µg/mm^3^, respectively. The water solubility in groups CC, 0 wt% hCS, 17.5 wt% hCS, 35.0 wt% hCS, and 52.5 wt% hCS were 6.11 ± 0.50, 13.87 ± 0.70, 14.06 ± 0.61, 22.36 ± 1.96, and 33.49 ± 1.68 µg/mm^3^, respectively.

### Ca and Si ion release

**Fig. 2:**
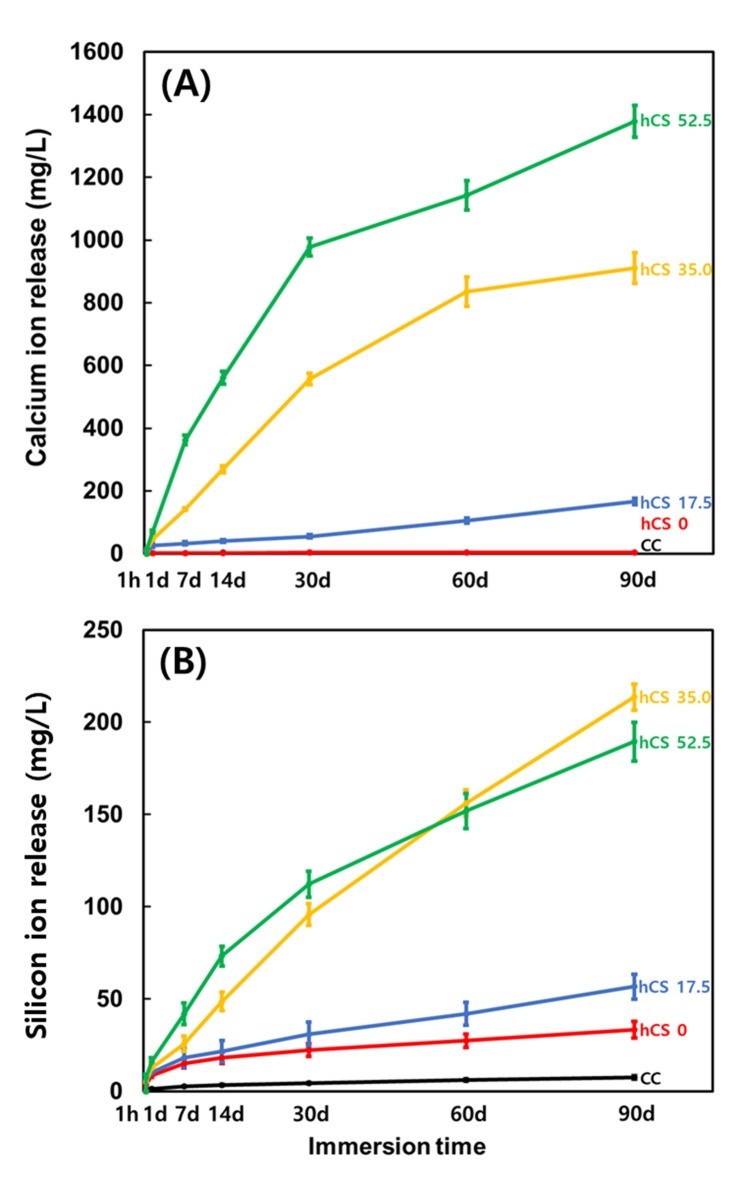
The Ca (A) and Si (B) ion concentration in the lactic acid solution at 1 h, 1 d, 7 d, 14 d, 30 d, 60 d, and 90 d. Each value shows the mean of six repeated measurements, and the error bars show the standard deviation of the mean values

Figure 2 shows the Ca ion release (A) and Si ion release (B) from the experimental groups at each time period. Accumulation of Ca ions was barely detectable in groups CC and 0 wt% hCS compared with that in the hCS-containing experimental groups at all-time points; the levels of accumulated Ca ions in the 17.5 wt% hCS, 35.0 wt% hCS, and 52.5 wt% hCS groups at 90 d was 165.96 ± 10.00, 911.55 ± 48.78, and 1378.12 ± 50.10 mg/L, respectively. Notably, groups 35.0 wt% hCS and 52.5 wt% hCS showed extremely rapid Ca ion release at 7 d; the cumulative Ca ion levels at 7 d from the 17.5 wt% hCS, 35.0 wt% hCS, and 52.5 wt% hCS groups were 33.18 ± 4.42, 141.96 ± 3.58, and 362.58 ± 15.26 mg/L, respectively. The release of silicon ions was lowest in CC, but higher than the Ca ion concentration of the same group, and the 0 wt% hCS group showed a slightly higher Si ion concentration than the CC group. Similar to the Ca ion concentration, the accumulated Si ion concentration presented a gradual increase as more hCS was added. In particular, groups 35.0 wt% hCS and 52.5 wt% hCS showed noticeably higher levels of Si ion release compared to group 17.5 wt% hCS. There was a rapid increase in Si ion release during 7 d, and the accumulated Si ion levels in groups CC, 0 wt% hCS, 17.5 wt% hCS, 35.0 wt% hCS, and 52.5 wt% hCS were 2.57 ± 0.23, 15.22 ± 2.51, 18.15 ± 5.14, 25.74 ± 4.12, and 42.17 ± 5.82 mg/L, respectively. The final cumulative Si ion concentration of CC, 0 wt% hCS, 17.5 wt% hCS, 35.0 wt% hCS, and 52.5 wt% hCS were 7.59 ± 1.00, 33.38 ± 4.52, 56.78 ± 6.72, 213.76 ± 7.07, and 189.55 ± 10.51 mg/L, respectively.

### Antibacterial effect

#### Evaluation of *S. mutans* attachment on the specimen by SEM

**Fig. 3:**
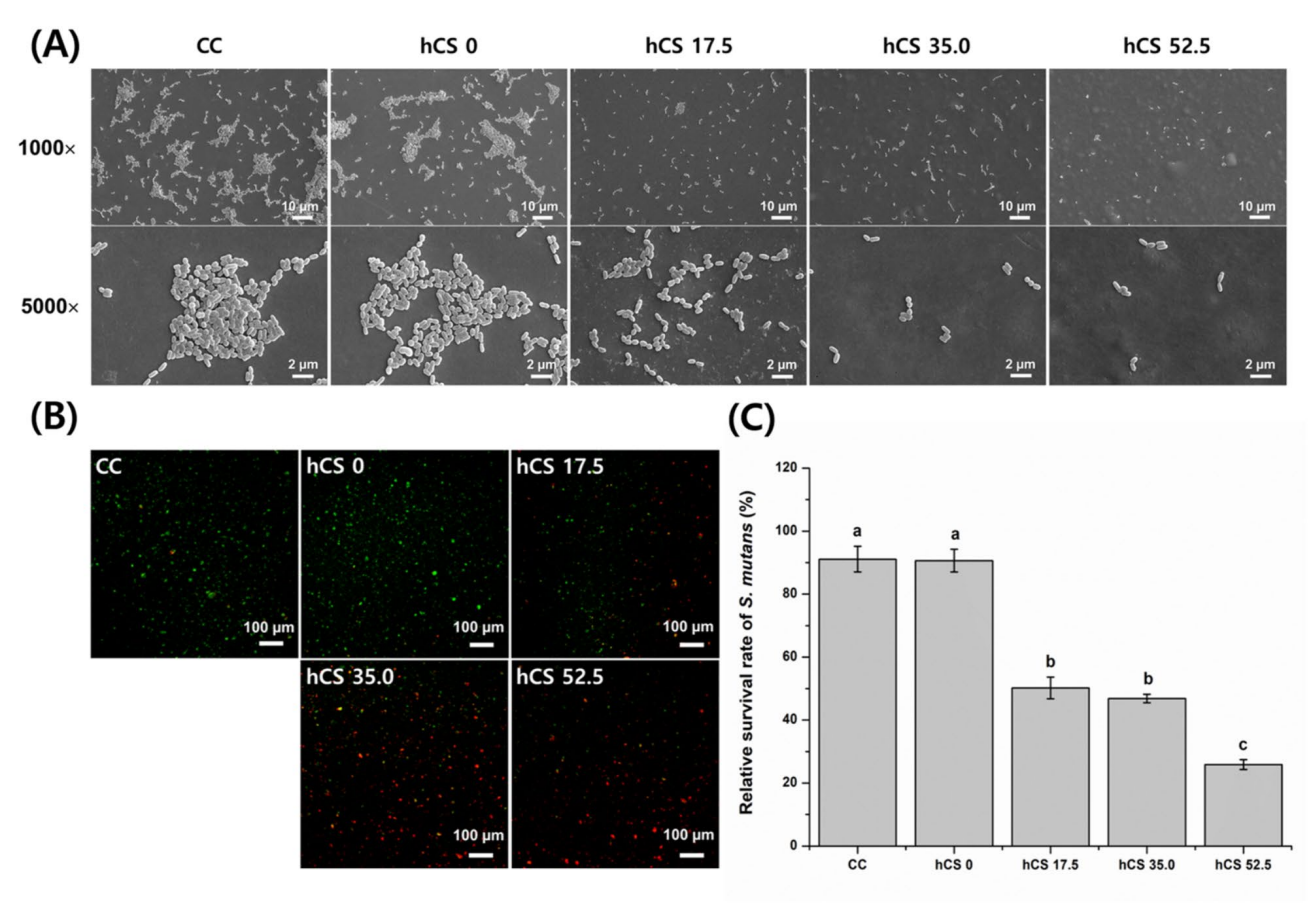
Representative scanning electron microscopy (SEM) images of *S. mutans* attached on the experimental resin surface with/without hCS at magnifications of 1000× on the upper, and 5000× on the bottom (A). Representative live/dead fluorescent staining images of *S. mutans* attached on the experimental resin surface with/without hCS using the confocal laser microscope. Green fluorescence indicates the presence of live cells, while red fluorescence indicates dead cells (B). Relative survival rate of *S. mutans* (%) by a quantitative analysis of CFUs on the experimental resin surface with/without hCS (C). The different letters indicate significant differences (*p* < 0.05)

Figure 3 A shows that *S. mutans* was attached to the specimen surface. The surface of CC showed densely adhered *S. mutans*, with the highest amount of *S. mutans* among all groups. 0 wt% hCS showed a lower amount of attached *S. mutans* compared to that in group CC and more than the hCS-added groups, but the aggregation of *S. mutans* was similar to that of CC. However, the adhesion of *S. mutans* decreased as the amount of hCS increased. In particular, it could be seen that 52.5 wt% hCS had lower distribution of the attached *S. mutans*.

### Bacteria viability by live/dead fluorescent staining

Figure 3B shows representative bacterial viability staining images of *S. mutans* on the surfaces. In general, CC and 0 wt% hCS were stained green, indicating that the bacteria were alive. In 17.5 wt% hCS, not only live bacteria but also dead bacteria that appeared in red staining were observed. On the other hand, visual examination confirmed that most of the 35.0 wt% hCS were dead bacteria. In addition, this aspect is more evident in 52.5 wt% hCS.

### Antibacterial activity by CFUs

Figure 3 C shows the relative survival rates of *S. mutans* (%) by quantitative analysis using CFUs. The relative survival rates of *S. mutans* decreased with the addition of hCS. They were significantly lower in groups 17.5 wt% hCS, 35.0 wt% hCS, and 52.5 wt% hCS than in groups CC and 0 wt% hCS (p < 0.05). There was no significant difference between the CC and 0 wt% hCS groups (p > 0.05). There was also no significant difference between the values of 17.5 wt% hCS and 35.0 wt% hCS (p > 0.05). 52.5 wt% hCS, showed significantly lower CFUs among all experimental groups (p < 0.05). The relative survival rates of *S. mutans* (%) in groups CC, 0 wt% hCS, 17.5 wt% hCS, 35.0 wt% hCS, and 52.5 wt% hCS were 91.10 ± 4.05, 90.63 ± 3.59, 50.21 ± 3.46, 46.83 ± 1.36, 25.87 ± 1.54, respectively.

### Apatite formation

#### SEM-EDS analysis

**Fig. 4:**
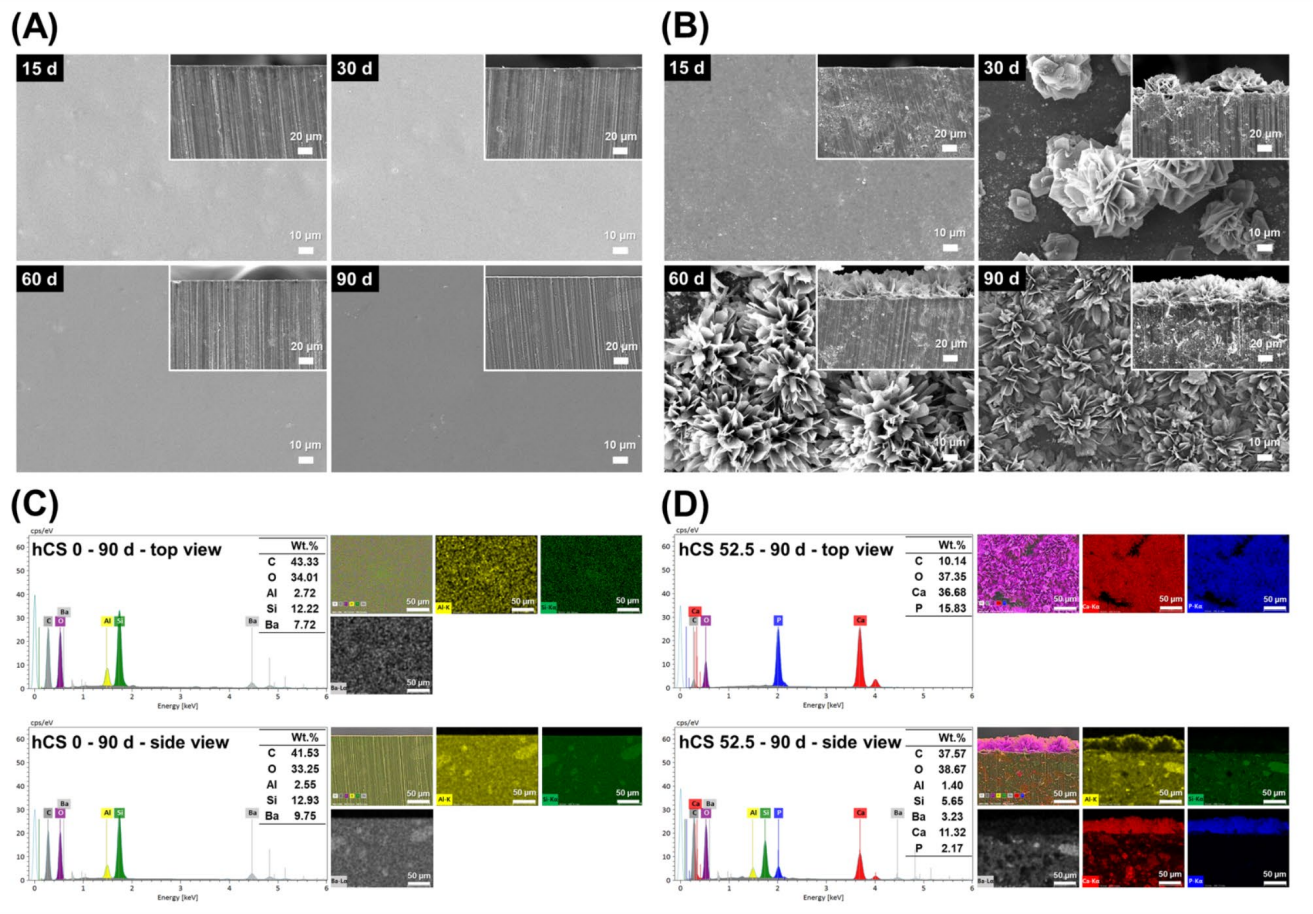
Representative SEM images of the 0 wt% hCS (A) and 52.5 wt% hCS (B) immersed in artificial saliva for 15 d (upper left), 30 d (upper right), 60 d (lower left), and 90 d (lower right) at magnifications of 500×. The main images show the surface of specimens, and the smaller images at the upper right are images of the cross sectioned specimens. Representative SEM-EDS images of upper and cross-sectioned surfaces of 0 wt% hCS (C) and 52.5 wt% hCS (D) immersed in artificial saliva for 90 d at magnifications of 500×. EDS results indicate Al as yellow, Si as green, Ba as grey, Ca as red, and P as blue

Figure 4 shows representative SEM-EDS images of 0 wt% hCS and 52.5 wt% hCS specimens. The surface images of the 0 wt% hCS specimens showed randomly spread spots which were micro and nanofillers, and there was no distinct difference between each time period (Fig. 4A). The dispersed micro and nanofillers were also detected in the cross-sectional image of 52.5 wt% hCS specimens (Fig. 4B). The surface and cross-sectional image of CC, 17.5 wt% hCS, and 35.0 wt% hCS specimens resembled those of 0 wt% hCS (data is not shown in Fig. 4). However, noticeable change was observed on the surface of 52.5 wt% hCS specimens after 30 d. Flower-shaped precipitates with a size of 50 μm appeared on the surface which then spread gradually on the surface over time. The SEM-EDS analysis of 0 wt% hCS specimens immersed in artificial saliva for 90 d showed Al, Si, and Ba as resin filler components (Fig. 4C). Unlike 0 wt% hCS group, Ca and P were detected in the 52.5 wt% hCS group, besides Al, Si, and Ba (Fig. 4D), while P was not detected in groups 17.5 wt% hCS and 35.0 wt% hCS (data not shown). Significantly, a thin layer on the top of the 52.5 wt% hCS specimens was found to be composed of Ca and P in the cross section image.

### Raman spectrometry

**Fig. 5:**
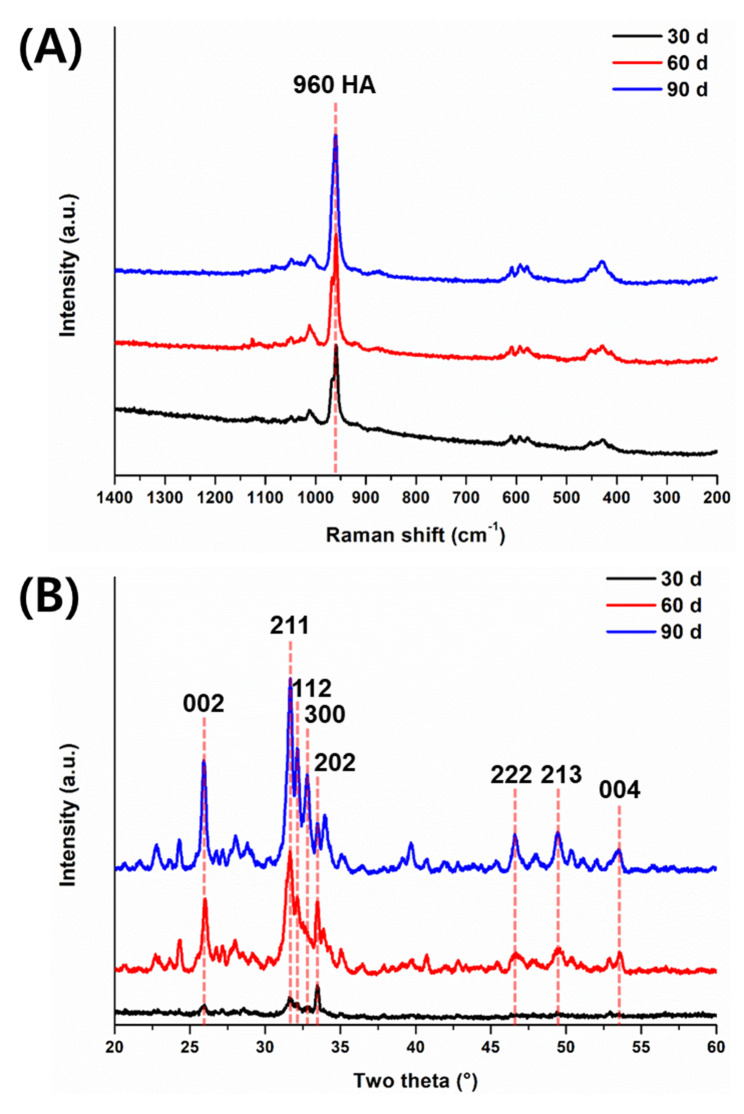
Representative Raman spectra (A) and X-ray diffractograms (B) of the precipitates on the 52.5 wt% hCS specimen surface after 30 d (black), 60 d (red), and 90 d (blue) of storage in artificial saliva. HA = hydroxyapatite.

The precipitates on the surface of the 52.5 wt% hCS specimens were carefully scraped, and then Raman spectrometry was performed. The Raman spectra of the 52.5 wt% hCS specimens immersed in artificial saliva for 30 d, 60 d, and 90 d are shown in Fig. 5A. Since no precipitate was formed on the surface of 52.5 wt% hCS immersed in the artificial saliva for 15 d, it was excluded for the Raman analysis. The intensity of hydroxyapatite at 960 cm^− 1^ was increased as the immersion period increased.

### XRD analysis

The XRD spectra of the precipitates on the 52.5 wt% hCS immersed in artificial saliva for 30 d, 60 d, and 90 d are shown in Fig. 5B. Since no precipitate was formed on the surface of 52.5 wt% hCS immersed in the artificial saliva for 15 d, it was excluded for the XRD analysis. The XRD spectra from 60 d and 90 d showed major peaks corresponding to the (0 0 2), (2 1 1), and (1 1 2) planes of hydroxyapatite. Carbonated hydroxyapatite was detected as (0 0 2), (2 1 1), and (2 0 2) planes after immersion in artificial saliva for 30 d, which can be overlapped with hydroxyapatite by similar d spacings [15]. The intensity of the detected peaks became evident as the immersion period was prolonged.

### Cytotoxicity analysis

**Fig. 6:**
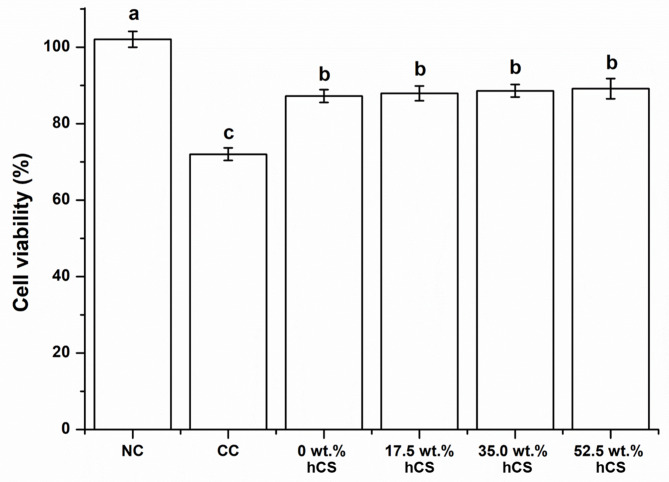
Cell viability of experimental groups. Each value represents the mean of the results, and the error bars represents the standard deviation of the mean. The same lower case letters indicate no significant differences among the groups (p > 0.05), while the different lowercase letters indicate significant differences between the experimental groups (p < 0.05). NC = Negative control, CC = Commercial control

Figure 6 shows the cell viability of experimental groups. They were significantly lower in groups 0 wt% hCS, 17.5 wt% hCS, 35.0 wt% hCS, and 52.5 wt% hCS than in groups NC (p < 0.05). There was no significant difference between the 0 wt% hCS and experimental groups containing hCS (p > 0.05). The lowest cell viability was observed in group CC (p < 0.05).

## Discussion

The effect of hCS has been extensively studied, especially in terms of dentin remineralization, pulp and bone regeneration, and bone/cementum tissue healing effects [16]. Despite the effect of apatite formation derived from calcium silicate-based cement on sealing ability, only a few studies have been carried out on self-healing restorative materials. White Portland cement and hydraulic calcium silicate-based cement have been used as substitutes for MTA in endodontic procedures [9]. Calcium ions released from calcium silicate-based cement interact with phosphate to form apatite-like precipitants, which generate mechanical and chemical bonds between the calcium silicate-based cement and dentine, and improve the sealing ability [8, 17]. Nevertheless, the use of hCS derived from white Portland cement is limited to endodontic treatment, and attempts to apply hCS to restorative materials are needed.

Dental composite resins are frequently used as restorative materials owing to their excellent esthetic and physiochemical properties. However, microleakage occurs due to wearing of resin, polymerization shrinkage, and breakdown of marginal integrity during function over time. In the present study, hCS was added to the experimental composite resin to generate a self-healing potential to reduce or seal microleakage at the interface of the restorative composites and cavity walls.

The properties of light-curable composite resin are largely affected by adequate light polymerization, and the un-polymerized composite resin may have decreased wear resistance and flexural strength [18, 19]. Consequently, it is important to ensure that the restorative resin has proper polymerization ability. The results of the depth of cure for each experimental group are shown in Fig. 1A. The CC group showed the longest polymerized length, and the results showed a decreasing tendency as more hCS was added to the experimental resin. However, it was still within an acceptable range of depth of cure according to ISO 4049. Therefore, adding hCS into the resin filler does not hinder the mechanical characteristic of the restorative materials.

To analyze the mechanical properties of the experimental groups, the three-point flexural strength of the specimen was measured. Mechanical properties are an important factor for the longer use of restorative materials [20]. Flexural strength is a fundamental factor that defines the rigidity and mechanical strength of the composite resin [21, 22], and resins with higher flexural strength are less likely to fail [23]. Figure 1B shows the flexural strength results of the specimens. The flexural strength value was highest for the CC group followed by the 0 wt% hCS group, and the strength increased with the addition of hCS to the experimental resin filler. According to ISO 4049, Type 2 restorative materials should have flexural strengths higher than 50 MPa, which indicates that all the experimental groups have clinically acceptable flexural strength. These results also support the results pertaining to the depth of cure that the addition of hCS does not restrain the mechanical properties of the experimental specimens.

Water sorption and water solubility can cause composite resin to undergo stress-induced degradation, which may cause microleakage [24]. The results of water sorption and water solubility of the experimental groups are shown in Fig. 1 C and D, respectively. Both water sorption and solubility showed similar tendencies: CC had the lowest value, while 0 wt% hCS and 17.5 wt% hCS were not significantly different (p > 0.05). In addition, water sorption and water solubility increased as more hCS was added to the experimental resin. Although an increase in water sorption and solubility could cause deterioration of the mechanical characteristic, it has merit as well. The swollen resin matrix may compensate for the stress caused by polymerization shrinkage [25]. Moreover, the flexural strength and depth of cure results showed that increased water sorption and water solubility did not hinder the flexural strength of the experimental groups.

The amount of calcium and silicon ions produced from the experimental specimens immersed in acid solution which simulated cariogenic environment is shown in Fig. 2. White Portland cement is primarily composed of tricalcium silicate and dicalcium silicate and hCS is known to release more Ca ions compared to CS [26]. Therefore, increased addition of hCS into the experimental resin could be resulted in higher calcium ion release. Silicon, a biologically important element, plays a key role in immunity [27, 28]. Groups without hCS showed significantly lower Si ion release compared to groups containing hCS, which may have resulted from the silanized glass filler. In contrast, as more hCS was added to the experimental resin, more Si ions were released, which implies that the experimental composite resin incorporated with hCS could have higher water solubility. In addition, the increasing accumulated concentration of released ions may have resulted in lower mechanical properties of the groups containing hCS, although still clinically acceptable.

Numerous studies used nano-silver, chlorhexidine, or other antibacterial agents on composite resins to prevent bacterial development for antibacterial benefits. Chlorhexidine which is frequently found in products to prevent oral infections attacks the outer and inner cell membranes resulting in cytolysis. Chlorhexidine can be added to dental composite resin, but doing so impairs its mechanical properties due to its immiscibility with monomers [29]. In addition, silver is known for its antibacterial effect and nano silver particles prevent bacterial development while maintaining the aesthetic properties of resin [30]. The issue with these antibacterial compounds is their early, fast release, which results in short-term effects. Therefore, improving the antibacterial properties of dental composite resins is critical for long-term durability [31]. One of the major cariogenic pathogens is *S. mutans* [32]. Thus, the most important strategy for overcoming the disadvantages associated with secondary caries is to inhibit *S. mutans* accumulation. The antibacterial effect of experimental materials containing different amounts of hCS was analyzed using both qualitative and quantitative analyses. As shown in Fig. 3, we found that the inclusion of hCS could inhibit bacterial viability. In qualitative evaluation, Fig. 3A and B show similar patterns in that fewer attached bacteria and the more dead bacteria were observed with an increase in the hCS content. In addition, these results were confirmed by quantitative evaluation by CFUs. As shown in Fig. 3C, the relative survival rate of 52.5 wt% hCS showed three times lower percentage compared with CC and 0 wt% hCS. In a previous study, high pH and calcium ion release from hCS were confirmed. It was also verified through the XRD results that calcium hydroxide as a by-product was included in the hCS particles [11]. Bacterial membrane proteins can be inactivated by a pH of 9.0 or higher [33]. In addition, it is also known that hydroxide ions have high antibacterial activity [34]. This mechanism involves hydroxide ions that can induce cell membrane lipid peroxidation and DNA damage [35]. In the present study, experimental groups containing hCS showed antibacterial effects compared to the CC and 0 wt% hCS groups. Therefore, the high pH and hydroxide ions produced by hCS might play an important role in antibacterial activity.

In order to evaluate the cytotoxicity of the restorative composite resin containing hCS, the MTT assay which measures the viability of cells through their metabolic activity was performed. In vitro studies evaluating the cell viability of restorative composite resin containing hCS showed a cell viability of more than 70% and only a slight growth inhibition effect. However, CC showed cytotoxic potential even though it was evaluated under the same experimental conditions. When assessing the safety of biomaterials, biocompatibility is a crucial factor to consider, and cytotoxicity is one of its important components [36]. Since dental materials should be biocompatible in order to be applied to the oral, there are several studies on the cytotoxicity of antibacterial dental restorations. The experimental materials in this research and the composite resin with 8% bioactive glass, both of which release calcium and silicon ions, demonstrated biocompatibility and minimal cytotoxicity [37]. In addition, resin with selenium-doped ZnO nanoparticles showed biocompatibility and advanced antibacterial effect [38]. Given that resin with either bioactive glass or ZnO was proven, the restorative composite resin incorporated with hCS demonstrated biocompatibility and could be a potential dental composite resin to be used in oral.

To analyze the apatite formation ability, the experimental specimens were immersed in artificial saliva. Some studies have applied specimens with various solutions, such as SBF, HBSS, DPBS, and distilled water. In this study, artificial saliva was used to reproduce the oral environment. SEM images of 0 wt% hCS immersed in artificial saliva for 15, 30, 60, and 90 d (Fig. 4) showed no particular difference as immersion prolonged, which could be considered as a control. These images can be observed in the general resin applied to the oral cavity. Compared to group 0 wt% hCS, flower-like cluster formation was appeared on the surface of 52.5 wt% hCS after 30 d, and it covered the surface of the specimens over time. The SEM-EDS images showed that Si, Ba, and Al are the components of the silanized glass filler, which were detected in both the 0 wt% hCS and 52.5 wt% hCS groups. However, for the 52.5 wt% hCS group, the precipitate layer formed on the surface of the specimens was confirmed to be composed of calcium and phosphate. Along with the released calcium ion results, abundant calcium ions from calcium silicate can be detected across the cross-section of the specimens (Fig. 4D). According to a previous study, calcium ions interact with the phosphate in the artificial saliva to form hydroxyapatite precipitates on the surface of the specimens [39].

Raman spectroscopy was conducted to analyze the precipitate layer, detected via SEM, on the surface of the 52.5 wt% hCS group. Raman spectroscopy is used in various fields because of its obvious spectral signals, which show differences in physical and chemical properties, and to identify both thin and bulk apatite [40, 41]. Raman analysis showed a stretching band from hydroxyapatite at 960 cm^− 1^ (Fig. 5A) [42]. The intensity of the detected hydroxyapatite peak at 960 cm^− 1^ after immersion in artificial saliva for 30, 60, and 90 d increased as immersion prolonged. In addition to Raman spectroscopy, XRD was performed to identify the precipitate layer on the surface. The XRD results showed carbonated hydroxyapatite peaks appeared on the surface of 52.5 wt% hCS after 30 d (Fig. 5b). The Raman and XRD results suggest that the precipitate layer formed on the surface of the 52.5 wt% hCS specimens was hydroxyapatite, which gradually piled up with prolonged storage in the artificial saliva. This could be supported by the SEM images showing hydroxyapatite formation with a growing number on the surface. In addition, the leveling off concentration of Ca and Si ion release suggests that the released ions were consumed to form hydroxyapatite.

Precipitates formed on the experimental resin containing hCS may break away from the resin surface when exposed to the mouth due to mastication with food and tooth brushing. However, deep microleakage between the restorative material and the teeth is difficult to reach when brushing, and the oral fluid circulation is less active, so precipitates may remain. Accordingly, deep in the microleakage area, hydroxyapatite precipitates made from the restorative material accumulate at the boundary over time, and there is a possibility that the gap can be closed. Along with the antibacterial effect, the aggregated hydroxyapatite could prevent secondary caries besides closing the microleakage. Silica nanoparticle is known for its biocompatible and when antibacterial agent is combined with the porous silica long-lasting antibacterial effect was demonstrated [43, 44]. In addition, the aggregated emission is known for its multifunctional properties including therapeutic effects [45]. These results from previous studies imply the hydroxyapatite formed in the microleakage could prevent secondary caries in both chemically and physically.

The potential of the composite resin containing hCS to form hydroxyapatite, as demonstrated by SEM, Raman, and XRD, suggests self-sealing ability under microleakage between the tooth and restorative material. Hydroxyapatite is the main mineral of enamel, and its chemical composition is similar to that of human hard tissue [46]. Therefore, the formation of hydroxyapatite on the surface of the experimental specimens implies sealing of the microleakage at the interface of the restoration and cavity walls with antibacterial effect. The limitation of this study is its high water sorption and solubility. However, since the 0 wt% hCS group, in which no hCS was added, showed even high water sorption and solubility, improvements need to be made through changes in the composition and proportion of resin matrix used in this study. In addition, further research is needed on the effect of restorative composite resin containing hCS on the gap in the oral environment when the materials are exposed to microleakage while being restored in teeth.

The limitation of this study is that materials containing calcium silicate should take their stability into account when they are subjected to wet conditions. Even though the restorative composite resin containing hCS showed low solubility in this study, more hCS in the restorative composite resin demonstrated higher solubility. Therefore, the long-term experiment under a wet environment could support its durability in oral conditions if it is used in practice. In addition, when dental product is used in practice, they should meet all regulations. It is challenging to market resin as a dental product, though, due to its characteristics alter during curing or even when administered orally. In this respect, even though the restorative composite resin with hCS is a promising resin, there is a variety of tests that should be conducted.

## Conclusions

This study examined the effect of a restorative composite resin containing hCS on the antibacterial effect and apatite forming ability to prevent secondary caries at the interface of restoration and teeth. The null hypothesis that the restorative composite resin containing hCS would not be significantly different in terms of mechanical and physical properties, ion release, antibacterial properties, and apatite formation from those of the composite resin without the hCS filler was partially rejected. The incorporation of hCS into the resin composite did not hinder the polymerization rate and flexural strength, even though water sorption and water solubility increased. Furthermore, it showed potential for antibacterial effects against *S. mutans* and hydroxyapatite formation ability. Consequently, composite resin containing hCS is a promising bioactive resin because of its clinically acceptable physiochemical properties, antibacterial properties, and self-sealing potential for prevention of microleakage for longer usage of restorations.

## Electronic supplementary material

Below is the link to the electronic supplementary material.

Supplementary Material 1

## Data Availability

The datasets used and analyzed during the current study are available from the corresponding author on reasonable request.

## List of abbreviations

DPBS: Dulbecco’s phosphate-buffered saline
HBSS: Hank’s balanced salt solution
hCS: Hydrated calcium silicate
MTA: Mineral trioxide aggregate
SEM/EDS: Scanning electron microscopy/energy dispersive X-ray spectrometry
SiC: Silicon carbide
SBF: Simulated body fluid
