# Supplementary material for: Novel antibacterial and apatite forming restorative composite resin incorporated with hydrated calcium silicate
Source: Biomater Res. 2023 Mar 29;27:25. doi: 10.1186/s40824-023-00364-z (PMC10053114; doi:10.1186/s40824-023-00364-z)
Supplement: Supplementary file 1 — Supplementary Material 1 [file 40824_2023_364_MOESM1_ESM.docx]

| Dear Editor and Reviewers of *Biomaterials Research*,  **Re: Revision of “Novel antibacterial and apatite forming restorative composite resin incorporated with hydrated calcium silicate” by Song-Yi Yang, A Ruem Han,** **Ji-Won Choi, Kwang-Mahn Kim, and Jae-Sung Kwon^*^ (BMRS-D-22-00322R1)**  We’d like to appreciate your kind reviews and comments regarding our manuscript (**BMRS-D-22-00322R1**) entitled above. Now we have carried out revisions according to your comments and hope this will be adequate for the acceptance of this manuscript. Details of corrections according to the comments are as follow; | |
| --- | --- |
| **Reply to reviewer # 1** | |
| Concern of the reviewer | Although the composite resin has demonstrated good antibacterial performance, it is suggested to test the cytotoxicity of the composite resin on normal cells. It is also suggested to compare the biocompatibility results with other antibacterial composite resins. |
| Our response | We appreciate your comments on this study. We strongly agree with your idea that a cytotoxicity test is needed as this material presents an antibacterial effect and externally communicates with the oral condition. Therefore, we assessed its cytotoxicity by an MTT assay and revised the paper accordingly. The detailed methods, results, and discussion are reflected in the “methods, results, and discussion” section (Page 8, 9, 13, 15, 16, 26, 27, Line 205-239, 343-347, 426-438, 684-687). Thank you once again for your input. |
| Revised text | **Methods**  **Cytotoxicity analysis**  **Cell culture preparation**  L-929 cells, a mouse fibroblast cell line, were cultured in 1X MEM (Welgene, Gyeongsangbuk-do, Korea) cell culture medium supplemented with 1 % antibiotic-antimycotic (Welgene, Gyeongsangbuk-do, Korea) and 10 % fetal bovine serum (Gibco, Grand Island, NY, USA). The cells were cultured in a humidified incubator at 37 ℃ with 5 % CO_2_. The adherent cells were separated with 0.05 % trypsin/EDTA (Gibco, Grand Island, NY, USA) and centrifuged for 3 min followed by resuspension in cell culture medium.  **Preparation of the samples**  Polymerized disk-shaped specimens (10.0 ± 0.1 mm diameter and 1.0 ± 0.1 mm height) from each experimental group were prepared. The sterilized disk specimens were extracted in a cell culture medium, 1X MEM containing 10 % fetal bovine serum, with an extraction ratio of 1.25 cm^2^/mL for 24 h at 37 ℃. The eluted 1X MEM containing 10 % fetal bovine serum for 24 h was used as a blank group and high density polyethylene film (Lot No.: C-212, Hatano Research Institute, Japan) was used as a negative group (NC). The L-929 cells were seeded into 96-well plates (SPL Life Science, Gyeonggi-do, Korea) and cultured in an incubator for 24 h at 37 ℃ with 5 % CO_2_. Then, the cells exposed to extracts solution were cultured in a humidified incubator at 37 ℃ with 5 % CO_2_. After 24 h, the extracts solution was removed and washed with PBS (Gibco, Grand Island, NY, USA).  **Methylthiazol tetrazolium (MTT) assay**  To evaluate the cytotoxicity of the experimental specimen, an MTT assay was performed according to ISO 10993-5:2009 (Biological evaluation of medical devices — Part 5: Tests for in vitro cytotoxicity) and ISO 10993-12:2021 (Biological evaluation of medical devices — Part 12: Sample preparation and reference materials). At a concentration of 1 mg/mL, the thiazoly blue tetrazolium bromide (Sigma-Aldrich, St. Louis, MO, USA) was dissolved in 1X MEM without phenol red medium (Welgene, Gyeongsangbuk-do, Korea). It was then filtered using a 2 µm syringe filter (ADVANTEC, Tokyo, Japan). The 50 µL of prepared MTT solution was added to each well, and the plates were cultured in an incubator at 37 ℃ with 5 % CO_2_. The MTT solution was removed after 2 h of incubation and 100 µL of isopropanol was added to each well. Then the absorbance was measured with a microplate spectrophotometer (Epoch, BioTek, Winooski, VT, USA) at 570 nm. The optical density (OD) was recorded and calculated following formulas to obtain the cell viability. Cell viability (%) = (OD_570_ of experimental group / OD_570_ of blank group) × 100  **Result**  **Cytotoxicity Analysis**  Figure 6 shows the cell viability of experimental groups. They were significantly lower in groups 0 wt.% hCS, 17.5 wt.% hCS, 35.0 wt.% hCS, and 52.5 wt.% hCS than in groups NC (p < 0.05). There was no significant difference between the 0 wt.% hCS and experimental groups containing hCS (p > 0.05). The lowest cell viability was observed in group CC (p < 0.05).  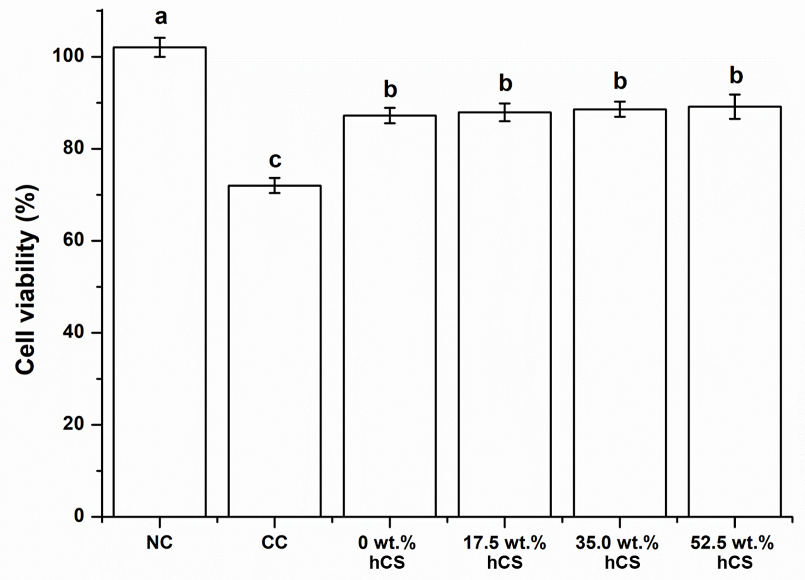  **Figure 6.** Cell viability of experimental groups. Each value represents the mean of the results, and the error bars represents the standard deviation of the mean. The same lower case letters indicate no significant differences among the groups (p > 0.05), while the different lowercase letters indicate significant differences between the experimental groups (p < 0.05). NC = Negative control, CC = Commercial control.  **Discussion – 8^th^ paragraph**  In order to evaluate the cytotoxicity of the restorative composite resin containing hCS, the MTT assay which measures the viability of cells through their metabolic activity was performed. In vitro studies evaluating the cell viability of restorative composite resin containing hCS showed a cell viability of more than 70% and only a slight growth inhibition effect. However, CC showed cytotoxic potential even though it was evaluated under the same experimental conditions. When assessing the safety of biomaterials, biocompatibility is a crucial factor to consider, and cytotoxicity is one of its important components [36]. Since dental materials should be biocompatible in order to be applied to the oral, there are several studies on the cytotoxicity of antibacterial dental restorations. The experimental materials in this research and the composite resin with 8 % bioactive glass, both of which release calcium and silicon ions, demonstrated biocompatibility and minimal cytotoxicity [37]. In addition, resin with selenium-doped zinc oxide (ZnO) nanoparticles showed biocompatibility and advanced antibacterial effect [38]. Given that resin with either bioactive glass or ZnO was proven, the restorative composite resin incorporated with hCS demonstrated biocompatibility and could be a potential dental composite resin to be used in oral. |
| Concern of the reviewer | It is better to quantitively to compare the antibacterial performance of the resin in this manuscript to other reported ones. Please briefly illustrate the pros and cons of different materials. |
| Our response | Thank you for the comment on this study. As you pointed out, this research will be more trustworthy if it compares different antibacterial resins to the resin used in this paper. According to your comments, “Discussion” was modified (Page 15, Line 403-409). Once again, I appreciate your guidance. |
| Revised text | **Discussion – 7^th^ paragraph**  Numerous studies used nano-silver, chlorhexidine, or other antibacterial agents on composite resins to prevent bacterial development for antibacterial benefits. Chlorhexidine which is frequently found in products to prevent oral infections attacks the outer and inner cell membranes resulting in cytolysis. Chlorhexidine can be added to dental composite resin, but doing so impairs its mechanical properties due to its immiscibility with monomers [29]. In addition, silver is known for its antibacterial effect and nano silver particles prevent bacterial development while maintaining the aesthetic properties of resin [30]. The issue with these antibacterial compounds is their early, fast release, which results in short-term effects. |
| Concern of the reviewer | It is better to cite relevant references such as Advanced Drug Delivery Reviews 2023, 192, 114634-114672; ACS Omega 2022, 7, 9, 7638-7647; Polym. Chem. 2021, 13, 8-43 |
| Our response | We appreciate your feedback on this research. The sources you gave are useful for discussing the antibacterial properties of the study's components. As a result, we expanded the discussion part to include a segment on the biocompatibility of this experimental material. According to your comments, “Discussion” was modified (Page 17, Line 471-477). |
| Revised text | **Discussion – 11^th^ paragraph**  Along with the antibacterial effect, the aggregated hydroxyapatite could prevent secondary caries besides closing the microleakage. Silica nanoparticle is known for its biocompatible and when antibacterial agent is combined with the porous silica long-lasting antibacterial effect was demonstrated [43,44]. In addition, the aggregated emission is known for its multifunctional properties including therapeutic effects [45]. These results from previous studies imply the hydroxyapatite formed in the microleakage could prevent secondary caries in both chemically and physically. |

| **Reply to reviewer # 2** | |
| --- | --- |
| Concern of the reviewer | Stability under wet environment is very important, especially when calcium silicate is considered. Despite authors demonstration of low solubility, have authors considered any disintegration of materials after prolonged water storage? Especially, as the results of the solubility increase with of the hCS filler content, this point would be important. |
| Our response | We appreciate your comments. We strongly concur with your idea that the materials with calcium silicate should be taken into account for their durability in an extended wet condition. We updated the text to reflect this restriction and note that we did not conduct an experiment involving long-term water storage (Page 18, Line 489-493). Again, thank you for your guidance. |
| Revised text | **Discussion – 13^th^ paragraph**  The limitation of this study is that materials containing hydrated calcium silicate should take their stability into account when they are subjected to wet conditions. Even though the restorative composite resin containing hCS showed low solubility in this study, more hCS in the restorative composite resin demonstrated higher solubility. Therefore, the long-term experiment under a wet environment could support its durability in oral conditions if it is used in practice. |
| Concern of the reviewer | The manuscript is scientific paper, where the novel material may still have limitation as the cement-based resin. This shall be stated in Discussion or Conclusions. A comparison with industrial composite resins may be ideal as these discussions will make a reader aware of the complexity required by real applications to patients. |
| Our response | We would greatly value your insightful advice regarding this research. As you pointed out, this should be noted as a limitation because dental products must adhere to a variety of standards before being used in practice. As a result, we adjusted the manuscript appropriately (Page 18, Line 493-496). We appreciate your remark once more. |
| Revised text | **Discussion – 13^th^ paragraph**  In addition, when dental product is used in practice, they should meet all regulations. It is challenging to market resin as a dental product, though, due to its characteristics alter during curing or even when administered orally. In this respect, even though the restorative composite resin with hCS is a promising resin, there is a variety of tests that should be conducted. |

Thank you once again for your review which significantly improved the quality of this manuscript.

Sincerely yours,

*Song-Yi Yang, A Ruem Han, Ji-Won Choi, Kwang-Mahn Kim, and Jae-Sung Kwon*
